# “Older people are weak”: perceptions and meanings of ageing and abuse against older people

**DOI:** 10.3389/fsoc.2023.1329005

**Published:** 2024-01-11

**Authors:** Isabel Dias, Silvia Fraga

**Affiliations:** ^1^Institute of Sociology of the University of Porto, Faculty of Arts and Humanities, University of Porto, Porto, Portugal; ^2^Institute of Public Health, University of Porto, Porto, Portugal

**Keywords:** ageing, abuse against older people, victimization, qualitative research, violence

## Abstract

**Introduction:**

This paper addresses the broader issue of elder abuse and seeks to analyse how victims and non-victims of abuse connect and explain the perception of ageing and the phenomenon of violence against older people.

**Methods:**

A qualitative study was conducted based on evidence gathered through 45 semi-structured interviews with people aged 60 or above who are part of the Portuguese EPIPorto population cohort. The interviews were analysed using grounded theory.

**Results/discussion:**

The results show that respondents link the ageist narratives that exist in our societies to the prevalence and naturalisation of violence against older people, that the risk of abuse increases with the weakening of family support networks, and that abuse is both a manifestation of asymmetrical power relations between victims and perpetrators and a severe violation of human rights. The findings also highlight the need to broaden the concept of violence against older people to include offences in the public sphere and not just in the context of the family and institutions.

## Introduction

The acknowledgement of elder abuse as a profound public health concern and a breach of human rights has undergone considerable progression (Yon et al., 2017). The World Health Organization states that elder abuse affects millions of people worldwide and has serious consequences at the individual, family and societal levels (WHO, 2022). It directly contributes to increased mortality and morbidity at this life stage (Pillemer et al., 2016). The abuse of older people continues to be a serious social problem and a risk to public health, estimated to be responsible for around 2,500 deaths per year (Mulroy and O’Neill, 2011; Sethi et al., 2011).

Most cases are invisible to society, take place privately, and are not reported to the authorities. The underreporting of abuse suffered by older people is therefore worrying. Social isolation, dementia, poor health and functional status hinder older adults’ ability to self-identify and report abuse. In addition, older people with cognitive or functional limitations may fear retaliation from a family member or formal carer, particularly in a home care setting. They may also feel unable to seek help, leading to underreporting and perpetuating abusive situations (Cannell et al., 2016). A previous study by Acierno et al. (2010) found that around 11% of older adults experience abuse. However, only 1 in 14 cases were reported to the authorities.

The underreporting of violence against older people, combined with a lack of consensus on the concept and how to measure it, makes it difficult to estimate its prevalence and assess the absolute magnitude of the problem (Yon et al., 2017). Despite these difficulties, the extent of the phenomenon generally varies between 3 and 30%, according to Mysyuk et al. (2016). Yon et al. (2017) estimated that one in six (15.7%) people aged 60 or above had experienced some form of abuse in the last year, based on a systematic review of 52 studies from 28 countries in different regions of the world. The authors noted, for example, that national estimates of elder abuse prevalence last year ranged from 2% to 6% in the United Kingdom, 4% in Canada, 18.4% in Israel and 29% to 30% in Spain.

The systematic review by Dong (2015) shows that the prevalence of abuse ranges from 2.2% to 79.7% and covers five continents, with significant geographical variations that may be due to cultural, social or methodological differences. This review also shows that the prevalence of elder abuse ranges from 10% among cognitively intact elderly to 47.3% among demented elderly. In Europe, prevalence ranges from 2.2% in Ireland to 61.1% in Croatia. In Asia, the highest 1-year prevalence in this review was found in older adults in mainland China (36.2%) and the lowest in India (14.0%). Only two studies were found in Africa, with prevalence ranging from 30 to 43.7%.

Studies also report different prevalence levels of abuse against older adults in European countries (Lindert et al., 2013), reaching 61.1% according to Aseem et al. (2019). In Portugal, the Abuel study (Soares et al., 2010) shows that the phenomenon has a prevalence of 27.4%, with older women (29.6%) being more affected than men (24.4%) (Fraga et al., 2014). The HARMED study (Dias et al., 2022) found that 23.9% of participants reported being victims of any type of abuse, with psychological abuse being the most common (19.9%). Among the different forms of abuse against older people, the literature has highlighted the prevalence of financial abuse (Mulroy and O’Neill, 2011), psychological abuse and physical neglect (Lindert et al., 2013).

Despite the difficulties of prevalence studies, they have been helpful, for example, in identifying the determinants of violence against older people and evaluating the effectiveness of social policies targeted at this population (Yon et al., 2017). However, elder abuse is a complex and multifaceted phenomenon, which is also characterised by the subjectivity of those involved (Mysyuk et al., 2016; Hackert et al., 2019). Grounded on this idea, this article aims to understand older people’s perceptions and meanings of this social problem. In pursuit of this objective, the study presents insights derived from comprehensive semi-structured interviews conducted with a cohort of 45 individuals aged 60 or older (Dias et al., 2022).

Overall, the article focuses on what victims and non-victims of abuse think about ageing in contemporary society, what abuse is, why it occurs, the most common types of violence, and their thoughts and feelings. Qualitative data from participants in the HARMED study (Dias et al., 2022) are used to validate these interpretations. Understanding their views on vulnerability and abuse in old age can inform effective and sustainable interventions to prevent and intervene in abuse (Cannell et al., 2016; Mysyuk et al., 2016).

## Literature review

Ageing is one of the research topics that has attracted the most interest from the international community of social scientists over the last three decades. One of the lines of research that has seen some development focuses on ageing as a vulnerability factor. It looks at identifying the factors that place older people at greater risk of social exclusion and violence (Pillemer et al., 2016). However, violence against older people as a research topic did not emerge until the 1980s (Daly et al., 2011). Although it was initially seen as an extension of domestic violence, the studies that have been carried out have focused on analysing both the prevalence of the phenomenon and the dynamics of the violence experienced by those aged 60 and older (Soares et al., 2010; Luoma et al., 2011; Fraga et al., 2013; Gil et al., 2015). Other studies have shown that there is also a link between socioeconomic inequalities (Fraga et al., 2014), lack of social and family support networks, poor general health and elder abuse (Melchiorre et al., 2013; Soares et al., 2013). Currently, there is concern about abuse as a violation of human rights (Clarke et al., 2016; WHO, 2022), as well as gender issues related to women’s longevity and the accumulation of experiences of discrimination and oppression throughout the life cycle (Valcore et al., 2021).

In fact, since the 1980s, research has paid attention to various factors that explain the phenomenon of violence against older people. Thus, according to Gholipour et al. (2020), theories have focused on describing perpetrators, studying victim characteristics, the relationship between victims and perpetrators, and analysing socio-cultural contexts. In summary, based on a review of available theories, the authors identify several factors that are at the root of the occurrence of violence against older people, namely the stress of the carer (situational theory); dependence, often mutual dependence between the older person and the perpetrator (social exchange theory); the economic problems, unemployment, lack of income, frustration and low education of elderly carers create stress and anxiety in the family (stratification theory); the negative attitudes towards older people and their exclusion from the labour market (political, economic theory); environmental stress, the inability of family members to cope with different social roles (role accumulation theory); learning and intergenerational transmission of abusive behaviour (social learning theory); caregiver pathology (caregiver psychopathology theory); domestic spousal abuse (feminist theory); finally, cultural values and social expectations towards older people (symbolic interactionism theory) (Gholipour et al., 2020).

This brief review of the main available theories shows that violence against older people is a multifactorial problem that requires the mobilisation of multiple approaches to explain the causes and risk factors of victimisation.^1^ It also indicates a need for an articulated multi-sectoral intervention to address this problem.

## Methods

### Study setting, research and design methods

The HARMED project is a cross-sectional study conducted in Portugal between January 2017 and July 2020 on the social, economic and health determinants of elder abuse in the context of an economic crisis. The project has been previously described elsewhere (Dias et al., 2022). In the first phase, a questionnaire was administered to 678 people aged 60 or above recruited from a population cohort in Porto, Portugal, the EPIPorto, which has followed 2,485 residents aged 18 and over since 1999 (Ramos et al., 2004). Also, as part of the HARMED project, a qualitative study was carried out to identify perceptions and feelings related to the experience of abuse through in-depth semi-structured interviews with 45 people aged 60 or above. An in-depth interview guide was developed, including questions about the status of older people in contemporary societies, violence against older people, the contexts and reasons for its occurrence, types of violence and perpetrators, and perceptions and feelings about the phenomenon.

### Sample and procedures

A purposive sampling approach was employed, based on the HARMED database and considering a diversity of respondent profiles. Drawing from the quantitative sample of the HARMED study (Dias et al., 2022), which encompassed 678 individuals aged 60 or older from the EPIPorto cohort of 2,485 Porto residents (Ramos et al., 2004) residing in the community rather than in institutional care, participants were selected to ensure diversity in sex, age, and experiences of violence. Inclusive criteria extended to participants who did not report any incidents of abuse.

Contact with participants adhered to the established protocol of the EPIPorto (ISPUP) cohort (Ramos et al., 2004). Eligible participants were initially sent a letter detailing the study, followed by telephone contact to schedule interview appointments. Throughout this process, efforts were made to achieve a balanced representation across all defined characteristics. Interviews were conducted in Portuguese by trained interviewers from the HARMED team between April and May 2018 at the research office of the University of Porto Medical School/University Hospital Centre of São João, a reference hospital in northern Portugal. Interviews were conducted at participants’ residences when mobility to the Medical School/University Hospital Centre of São João was impeded due to health or functional constraints.

Written informed consent, with guarantees of confidentiality and anonymity, was obtained from the participants. All participants gave their consent for the interviews to be audio-recorded. Participants were contacted in order to get a balanced sample for all the characteristics previously defined. A sample of 20 men and 25 women, aged between 60 and 85, was identified reached. Recruitment stopped when theoretical saturation was reached.

The main focus of the interview guide was on social representations of the phenomenon of violence against older people. The guide included questions about perceptions of ageing (e.g., what do you think it means to be older nowadays? What do you think about the way older people are treated today? How do you think families treat old people today?); and the phenomenon of violence against older people, how older people define violence and its types, contexts and reasons for its occurrence, feelings about the phenomenon (e.g., what is your understanding of abuse against older people? What situations do you consider to be abuse? What do you think is the most serious type of violence that can be perpetrated against an older person? Where do you think the abuse of older people most commonly takes place? Who are the most common aggressors of older people? What do you think people feel when they are victims of abuse?).

The formulation of the questions to be included in the interview guide was based on the literature review but also on the theory of intersectionality (Atewologun, 2018). The main purpose was to formulate questions that allowed us to understand how older people perceive vulnerability and the accumulation of vulnerabilities among older people as one important determinant that increases the risk of abuse. The theory of intersectionality led us to consider participants’ voices as the central role and to interpret the information gathered in relational, social and political contexts (Atewologun and Mahalingam, 2018). The final guide was adapted to the specific characteristics of the participants and the study objectives of the HARMED project. The questions were pre-tested, and a preliminary content analysis of the pre-test interviews was carried out to select the questions that mostly demonstrated to answer the project objectives. The duration of the interviews ranged from 30 min to 3 h.

The joint ethics committee of the S. João Hospital and the Medical School of the University of Porto approved this study protocol (CES-320/2016). The WHO ethical safety guidelines for research on violence were followed (WHO, 2001). A manual describing and adapting these guidelines was prepared to be used by interviewers.

### Data analysis

The analysis of the interviews was based on the grounded theory approach (Glaser and Strauss, 1967). The aim was to identify a set of categories of properties and relationships between the data. Open inductive coding was used, allowing comparison of the data, leading to new codes for emerging themes and the generation of interpretations based on the data (Cannell et al., 2016; Heydarian, 2016). After organising the information into codes and possible themes, thematic content analysis was applied (Braun and Clarke, 2006; Heydarian, 2016). The final validation was done through inter-judge agreement, that is, through discussion and confirmation by the authors that the themes are consistent given the codes identified. This type of analysis gives validity to the data collected and meaning to the context in which it was collected, thus contributing to a better understanding of the information from the interviews (Martins et al., 2021).

This method of analysis allowed us to explore the different ways in which respondents, through the verbalisation of attitudes, shared their perceptions and feelings about violence against older people. It also allowed us to deepen some of the HARMED survey themes and identify new issues that concerned respondents.

## Findings

This section of the article presents the characteristics of the interviewees and then reports the findings on the following categories to which thematic content analysis was applied: status of older people in contemporary societies; explanations, definitions, reasons and feelings about elder abuse. The respondents’ statements on the analysed thematic categories support the findings.

### Characteristics of the qualitative study participants

As mentioned above, the sample participating in the interview situation includes 20 men and 25 women aged between 65 and 87. More than half of the sample has a level of education that is divided into low (< 5 years) and medium (5–9 years), and the other half has a high level of education (> 9 years). They are predominantly married (73.3%) and widowed (20.2%). The rest are single or divorced (6.6%). The occupational status of the respondents is mainly retirement. Only 15.5% are still working.

Regarding experiences of abuse, 27 of the 45 respondents (60.0%) reported being victims of some form of abuse in the past 12 months. The most common type of abuse was psychological abuse (46.7%). This was followed by financial abuse (20.0%), as shown in Table 1.

**Table 1:** Demographic characteristics and types of abuse experienced by participants in the interviews (HARMED Qualitative study, *n* = 45).

Demographic characteristics	*n*	%
**Sex**		
Male	20	44.4
Female	25	55.6
**Age (years)**		
60–64	8	17.8
65–74	21	46.7
75–84	14	31.1
≥ 85	2	4.4
**Education level**		
High (>9 years)	17	37.8
Intermediate (5–9 years)	12	26.7
Low (<5 years)	16	35.6
**Marital status**		
Single	2	4.4
Married	33	73.3
Divorced	1	2.2
Widow	9	20.0
**Occupational status**		
Retired	35	77.8
Full-time job	7	15.6
Unemployed	1	2.2
Retired on invalidity	2	4.4
**Any type of abuse in the last 12 months**		
Yes	27	60.0
No	18	40.0
**Type of abuse**		
Psychological	21	46.7
Physical	7	15.6
Financial	9	20.0
Sexual	3	6.7

### Ageism, illness and discriminatory attitudes: being old in today’s society

Regarding the place of older adults in contemporary societies, respondents were divided between optimistic and pessimistic discourses on ageing.

The optimistic view is linked to the greater longevity of older people, family life, and the possibility of having more time for oneself.

*“With the increase in life expectancy, I think an elderly person today is 75–85 years old and above.”* (Male, 65–74 years, victim of abuse).

*“People tend to live longer, medicine is very advanced, and it is easier to treat themselves. Today’s diet will also be different.”* (Male, 65–74 years, victim of abuse).

*“I believe that ageing is a phase of life that is also good in that we are more available for our children and grandchildren. We no longer have this routine work (…). Old age is also good because we can think. I like to have free time to think and let my mind wander. I feel that when you are retired you can do certain things that you like.”* (Female, 75–84 years, non-victim).

Aspects such as ageism, sickness and the discrimination they suffer in our societies are the main negative discourses on ageing highlighted by respondents.

*“Older adults today are treated like rubbish, like useless people. They have no power to organise or control entitlements; society does not use them. They take pills. (…) Older people could do so much for society, like part-time jobs, organising libraries, helping in hospitals, and helping the sick. There are so many things that older people can do.”* (Male, 65–74 years, victim of abuse).

*“I think that society sees older people as useless. They think that older people are just a piece of meat that runs around and makes life difficult for others.”* (Male, 60–64 years, victim of abuse).

*“We mistreat older people! They are alone, without any support. It’s even worse in big cities. Today, the elderly person does not have a place in the family. It’s more of an obstacle than anything else.”* (Female, 60–64 years, victim of abuse).

Associated with ageist representations of ageing in the discourse of respondents is also a view of ageing as a time of illness and dependency.

“*Everything helps you to get older, time goes by, and then there are illnesses that appear. Some worries are also part of the ageing process. My wife is also quite ill. She suffers a lot too, she’s very nervous and upsets me, which makes me grow old. It is not easy*.” (Male, 65–74 years, victim of abuse).

*“I feel that I am getting older. I want to live near my family because I might need some support. I have lived in Porto (Portugal) for over 50 years, but here, I notice that the world differs from when I was young. People do not care about other people anymore. That’s why I’m looking for a house closer to my nephew. My nephew will help me if I suddenly need something.”* (Female, 75–84 years, victim of abuse).

*“We have lost the abilities that we used to have. For example, since I was 80 years old, I’ve lost a lot of my mobility, my eyesight is impaired, I have no teeth, I cannot walk much, and I have breathing problems. I mean, these are the problems that come with old age.”* (Male, 75–84 years, victim of abuse).

Therefore, illness, motor disability and death are the characteristics of old age identified by respondents:

*“Some of the people I think of as old are the ones who already have Alzheimer’s disease or dementia.”* (Female, 75–84 years, non-victim).

*“I go to bed at night, I go to sleep, and I think, am I going to wake up tomorrow? It’s this feeling. It’s the thought of death.”* (Male, 65–74 years, non-victim).

### Views and explanations of abuse: individualistic society and abuse as a crime

Respondents see violence against older people as a serious social problem comparable to other forms of domestic violence, such as violence against women and children. They generally condemn violence against older people and see it as a reflection of our society and an intergenerational problem.

*“Society is more selfish, in my opinion. People live more for themselves. They do not care much about what happens to others. I think society has encouraged violence against older people in this respect. The history of ‘every man for himself’, first me, then others, so I think older people have lost a lot in society.”* (Male, 65–74 years, victim of abuse).

*“The blame for all this is also a bit of our generation, the generation I belong to, because it was a generation that had values that are there now, for example, the selfishness, the lack of education of people.”* (Male, 65–74 years, victim of abuse).

Older people are also seen as more vulnerable to abuse due to the weakening of family relationships, lack of respect, and lack of communication with younger generations.

*“The families are dysfunctional; then they end up taking it out on the older adults; there is a lot of frustration: I do not have the time; I do not have to take care of my parents. All this has an impact on the family relationship.”* (Male, 65–74 years, victim of abuse).

*“When I was a child, the elderly were highly respected because the concept of family was much closer than it is now. In my house and among the close people I lived with, there’s a much stronger concept of family. Families were big. There were always grandparents, elderly uncles, who were respected and gave a certain balance to relationships.”* (Male, 60–64 years, non-victim).

*“You cannot talk to young people. Older people only talk about ‘my time, my time’. People are disconnected. The world I was born into has nothing to do with today’s world. Nothing! And people do not have the patience to put up with an old man any more.”* (Male, 75–84 years, non-victim).

Respondents also mentioned that violence against older people is a manifestation of the power asymmetry between the aggressor and the older victim. Another reason for abuse in the respondents’ discourse is the decline in physical abilities and the increased likelihood of illness due to biological ageing.

*“The stronger ones tend to use that power to hurt physically and psychologically. Older people are weak; they are diminished in front of someone who has more power than the other, so they treat older people badly.”* (Male, 60–64 years, victim of abuse).

*“There is much more violence against old people because they are more vulnerable.”* (Male, 65–74 years, non-victim).

*“The incapacity of the elderly person has a lot of influence. The weaker you are physically, the less able you are to defend yourself, and the easier it is for you to become a target.”* (Male, 65–74 years, victim of abuse).

In explaining violence against older people, respondents defined abuse as a crime and a violation of human rights.

*“Violence is a crime. It is not just against older adults. It is against older adults, it is against young people, and it is against everything and everyone. Whatever it is, be it domestic violence, be it sexual violence, be it any violence. Violence is a crime.”* (Male, 75–84 years, non-victim).

*“If there are more attacks, it’s a public offence. The law clearly states that it is a public offence. People should complain so that society itself realises that these situations should not go unpunished. We can no longer accept the popular saying that no one can interfere between a man and a woman.”* (Male, 60–64 years, victim of abuse).

The perception that violence against older people is a serious problem is shown when those interviewed say that violence is a violation of human rights: *“Any kind of violence is always a massive lack of respect for the person. The human person is the most dignified and noble being on earth. We must remember that human frailty comes with time. Even if a person has lost the qualities he had when he was working, he is still a human being. For this reason, the human being should be respected in the totality of their weaknesses.”* (Male, 75–84 years, non-victim).

### Perceptions and definitions of abuse of older adults

The definitions of violence against older people as they appeared in the speeches of the interviewees are presented in this section. The aim was to keep the concept of violence against older people open enough to accommodate and classify the interviewees’ perceptions and experiences of violence. Although there are several definitions (Castle et al., 2015), in the Harmed study (Dias et al., 2022) we use the WHO (2002) definition of elder abuse: “Elder abuse is a single or repeated act, or lack of appropriate action, occurring within a relationship in which there is an expectation of trust, that causes harm or distress to an older person” (p. 3). It is defined as misbehaviour towards older people by those in a position of trust, power or responsibility for caring for older people (Gholipour et al., 2020). The concept encompasses different types of abuse: physical, psychological/emotional, sexual, financial and neglect (WHO, 2002; Yon et al., 2017, 2019; Phelan, 2018; Naderi et al., 2019). It is currently considered a violation of rights (Clarke et al., 2016; WHO, 2022), denial of privacy and participation in decision-making (Gholipour et al., 2020).

Abuse of older persons is further classified by the type of perpetrator—family members, informal and formal caregivers or acquaintances—or by the context in which it occurs—in the community, residential care facilities, and nursing homes. In residential care settings, elder abuse can be categorised as resident-on-resident or staff-on-resident abuse (Yon et al., 2017, 2019). This article also explores respondents’ perceptions of the behaviours they consider to be abuse by professionals or formal carers in residential care settings (i.e., nursing homes, assisted living facilities, long-term care facilities and sheltered housing) (Castle et al., 2015; Malmedal et al., 2020).

The scientific definitions of elder abuse and the respondents’ definitions in their own words are presented in Table 2. As mentioned earlier, the main aim of this table is to compare the scientific definitions of elder abuse with the perceptions of those interviewed with and without experience of abuse as to what abuse is.

**Table 2:** Definitions of types of abuse by respondents’ victimisation status.

Scientific definitions^a^	Respondents’ definitions	
	Victims	Non-victims
**Psychological abuse** [Actions leading to psychological, emotional and mental distress]	*Imposing behaviour* *Boxed into rubbish bin* *Hurting people in a verbal way* *Not having freedom* *Pretending to be a rag* *Family members leaving them to their fate* *Not looking at the old person in the street* *Saying: “You’re no good, you’ll never die”* *Saying: “Do you know where you were good? It was at home, sleeping”* *Saying: “You are here too much”*	*The person who is cast aside* *People in nursing homes are abandoned, waiting for the time of death to come.* *Saying: “My old man will never die.* *Saying: “You’re useless, you cannot do anything anymore.”* *Saying: “You’re useless; you cannot do anything anymore”*
**Physical abuse** [Acts that cause pain and physical injury]	*Shooting* *Stabbing* *Pinning to chairs* *Husband beating wife* *Beating with instruments—sticks, guns, knives* *Drugging and overmedicating* *Beating to death*	*Assault* *Forcing a person* *Tie to bed*
**Financial abuse** [Misappropriation of the resources, assets and property of an older person]	*Control of money by the husband* *Being robbed on public transport* *Inheritance and property division robbery* *Having an interest in the savings of older adults* *Sons do not look for ways of getting money* *Abandonment when older people do not have money* *Children’s attachment to their mother and father is a function of income.*	*Older adults are treated as animals if they do not give their children and grandchildren money.* *Taking care of parents to enjoy any goods or property* *Murder of mother for pension money for drug abuse*
**Sexual abuse** [Sexually explicit acts or sexual activity without the older adult’s consent]	*Rape of women* *Husband forces wife to have sex*	*The rape of women*
**Negligence** [Failure to meet the needs of older adults and putting their well-being at risk]**& Abandonment** [by the person responsible for the elderly person’s care or by a carer’s imprisonment]	*Not having help with hygiene,* *mobility needs and meals;* *Lack of affection, tenderness and understanding* *Abandonment at the nappy stage* *Not helping a person who falls in the street* *Being segregated or pushed aside* *Abandoned in hospital*	*Not paying attention* *Turning your back on the elderly* *Lack of access to food and medicine* *Not caring for an older person*
**Abuse in institutional settings** [Abuse perpetrated by professionals or formal carers in residential long-term care facilities, i.e., staff to resident abuse]	*Being stuck in a chair in a nursing home* *Killing with excess pills* *Old people are treated badly in nursing homes.* *Filling older adults with pills*	*Nursing homes are not equipped with technicians who can give quality of life* *They are beating the old people in the institutions* *A deposit for dying*

a Pillemer et al. (2016); Naderi et al. (2019); Yon et al. (2017); Gholipour et al. (2020); Castle et al. (2015); Dias et al. (2020).

Table 2 identifies participants’ perceptions of what constitutes abuse and the main types of abuse. People who have been abused are more familiar with the different ways abuse can occur. They describe abuse in detail, give examples and recount situations in which they have been victimised. Participants who have not been victims do not describe the different types in the same detail, but some knowledge of the phenomenon is evident in their statements. This knowledge is mainly the result of information from the media.

The following statements illustrate respondents’ perceptions and shared knowledge in this area. They also indicate the severity of each type of abuse as perceived by those interviewed.

*“Violence can take many forms; it can be physical or psychological. Sometimes the psychological violence ends up being worse than the physical violence. Still, violence is everything that can be done to other people without them even being able to defend themselves.”* (Male, 65–74 years, non-victim).

According to the respondents, psychological abuse is a type of violence that causes psychological and emotional pain. It is a long-term process often not recognised by the victims as a form of violence.

*“It’s the one who’s going to get dumped.”* (Male, 75–84 years, victim of abuse).

*“It’s the insults, for example, treating a grandmother with an insult, “look, go here, go there,” I think it’s something like … for me it’s like cutting with a knife*.” (Female, 75–84 years, victim of abuse).

*“Physical violence, of course, we all know that there are marks on the body, but emotional violence is more serious because the effects do not go away quickly.”* (Female, 65–74 years, victim of abuse).

However, physical abuse is seen as the most serious but also the easiest to identify, as two of the respondents pointed out:

*“More serious, I think, is when they beat older people! They’re beaten because they cannot defend themselves if they are mistreated. A person who already has a low standard of living does not have the strength for a fight, does he?”* (Female, 60–64 years, victim of abuse).

*“We hurt older people a lot verbally, but I think physically it is even worse (…) physically, I think no one has the right to hurt anyone, especially an older person.”* (Female, 75–84 years, victim of abuse).

Physical violence is the final stage in the cycle of violence for other respondents: *“By the time you reach aggression, you have already gone through many steps, from insulting the person, mistreating them, not caring about them. And then it ends with aggression. And you reach the height of violence when you get to the point of aggression.”* (Male, 65–74 years, victim of abuse).

Physical violence is often used as intimidation, which helps perpetrators carry out financial abuse. According to respondents, this is also very common among older people: *“I think that often the physical violence against older people is also to exploit them economically. I’m not sure, but that’s how it seems to me.”* (Female, 75–84 years, non-victim).

A material interest in the inheritance and property of older people, in the opinion of respondents, leads to financial abuse of parents at this stage of the life cycle by relatives such as sons and daughters-in-law:

*“Sometimes it’s for material interests, ‘look [daughter-in-law], your father could give us this or that or loan us money, but he does not want to, he’s a crook’ (…) it’s that kind of talk.”* (Male, 75–84 years, non-victim).

*“There are some good children, but there are also other children who just want to get money for their pension and who abuse their parents.”* (Female, 65–74 years, victim of abuse).

*“Children only visit their parents to get money. Otherwise, the parents are not good for anything else.”* (Male, 75–84 years, non-victim).

*“The older people have children who want money for drugs, money to get drunk and all sorts of things. The parents, the poor things, and the older people they say that they will not give them any money, and then they kill them.”* (Female, 65–74 years, victim of abuse).

Neglect is mentioned with great concern by both victims and non-victims as a very common form of abuse:

*“Neglect means that we [the elderly] are mistreated regarding food, hygiene, health, everything.”* (Female, 65–74 years, victim of abuse).

*“Do not give them the attention they need and deserve (&) do not feed them, all this is violence against older people.”* (Female, 65–74 years, non-victim).

*“The mistreatment, it’s the way you see her leaning over, not holding her hand, falling to the floor. “Who is it?” Oh, let him be.”* (Male, 75–84 years, non-victim).

According to interviewees, neglect manifests itself in a lack of food and medicine, a lack of help with hygiene and mobility, affection, understanding and visits from family and friends, or even a lack of support in daily life.

The violence least mentioned by the interviewees is sexual abuse. Only one man referred to it as the practice of *“raping women”* (65–74 years, non-victim). Some women interviewed are also very reluctant to discuss this type of abuse. However, one woman defined it as *“non-consensual acts, such as being forced to have sexual intercourse by the husband or strangers.”* (Female, 65–74 years, victim of abuse). Despite the participants’ reluctance to broach the subject, one interviewee admitted to being a victim of sexual abuse at the hands of her husband: *“Yes, he [husband] wanted to abuse me, but I did not let him. I do not like this [sexual] work. I’m old. I’m not modern for these things. I’ve always said no. I’ve said it to the police and in court (…). My husband is an old man, and these older men are too much; they are nothing like today’s youth; they are the worst.”* (Female, 65–74 years, victim of abuse).

Another respondent said she had been sexually abused as a child: *“When I was a girl I was abused by a neighbour and out of shame I never told anyone, not my husband, not my children. I never said anything. I happened to get on well with his daughters, we played together. I never told. I was afraid and ashamed. This trauma remained. I cannot forgive him. God forgive me. They say it’s a disease, but I cannot!.”* (Female, 65–74 years, victim of abuse).

The results show that some respondents perceive sexual abuse as one of the most serious forms of abuse: *“For me, raping an elderly person is the worst; it’s the worst of all forms of violence, and the abusive words they [the perpetrators] say, I heard them on TV once, besides the lady, poor thing, being raped, the abusive words they say.”* (Female, 65–74 years, victim of abuse).

Finally, abuse in long-term residential care institutions is also mentioned by respondents. This is known as institutional abuse, perpetrated by people paid to provide care and services to older people. It manifests in various ways: excessive restrictions, under-or over-medication, physical abuse, threats, degrading language, intimidation, verbal aggression, psychological, sexual and financial abuse, infantilisation, depersonalisation, and so on (Castle et al., 2015; Pillemer et al., 2016; Dias et al., 2022).

*“It pains me to look at old people’s homes and see them waiting for the day, the hour, being pushed and fenced in. (…) The old adults go to a kind of warehouse to die. Of course, the state has a role in subsidising and supervising. The sooner they die in nursing homes, the better; the sooner other older adults come, the better. I think there is a lack of love, affection, tenderness and understanding in older adults’s homes. I think these structures should be equipped with health technicians aware of all the circumstances. The state should severely punish all these situations.”* (Male, 75–84 years, non-victim).

*“I know that they beat the elderly in the institutions. It’s a shame if you are a visitor to an old people’s home.”* (Male, 65–74 years, victim of abuse).

*“We go to a nursing home; we are treated by people who are nothing to us if they do not have a good education. What if I go to a home where those people (formal caregivers) do not have a love for me, no affection? It’s not like me with my mother, who has respect for her. The news we hear is that if a person goes to a nursing home and says yes to everything, everything will be fine; if we are a bit more stubborn and think that this or that is not right, what do they do to us? They fill us with pills so we can sleep there, and after a short time, a person goes from this to that.”* (Female, 65–74 years, victim of abuse).

The idea that residential care homes for older people provide opportunities for abuse is present in the participants’ discourse. This perception is reinforced by the fact that older people in care homes are more likely to have multiple forms of disability, including mental, physical or behavioural abnormalities, as well as disabling conditions. Thus, because of their frailty, residents in institutions tend to be more dependent on others for care and may be at greater risk of abuse than older people in community settings. Finally, the prevalence of abuse may be much higher than reported. This under-reporting may be due to victims’ inability to report the abuse or fear of repercussions and retaliation (Yon et al., 2019).

### Why are older adults vulnerable to abuse?

It is increasingly recognised that vulnerability in old age results from complex interactions between risks, exposure to threats, their occurrence and/or lack of resources to cope with these threats and risks (Grundy, 2006). Thus, vulnerable older people are those whose resource reserves are below the necessary threshold or may even be depleted. These may be physical and mental health, social and family networks, material resources, legal and moral rights, coping strategies, personality, social abilities, gender status, inter-generational and community solidarity. The lack or inadequacy of these resources can make it difficult for older people to meet the special challenges of ageing (Grundy, 2006; Clegg et al., 2013), including coping with the abuse they may face.

According to the interviewees, one factor that makes older people vulnerable to violence is the imbalance of power between perpetrators and victims. In their discourses, the perception of older people as fragile and helpless is very present, and they feel like they were returning to childhood. Thus, ageist representations gain ground among the interviewees, leading them to associate vulnerability to violence with the infantilisation of older people at this life stage.

*“The strongest have a tendency, either physically or psychologically, to use that power to hurt; older people are weak, they are diminished, and someone with power, with a little more power, abuses them. Violence is always done in the same way; when one person wants to hurt another and feels stronger, he abuses and does it. Because old people are old, they are like children. Because they have less strength, there is abuse.”* (Male, 60–64 years, victim of abuse).

*“Violence against older people is an abuse of the weakest members of society.”* (Female, 60–64 years, victim of abuse).

*“The old people are more defenceless people; I already have a certain reserve because I know that at my age, I feel more defenceless by nature; I do not have as much strength.”* (Male, 65–74 years, *non-victim*).

*“The elderly are more fragile. They (the perpetrators) take advantage of this.”* (Male, 65–74 years, victim of abuse).

Older adults are equally vulnerable to violence because of their lack of family and financial support, their dependent status, and the physical limitations common in the advanced stages of the life.

*“Some old people are abandoned and you ask yourself: “How is it possible to do this? Does not this person have a family? That’s what I say. The 200 euro pensions are not enough. People have no support, in other words, the elderly have no support. These are the cases we see on television. An 85-year-old man was abandoned, in another community a 90-year-old man was abandoned and another died alone at home. This is all part of today’s society.”* (Male, 65–74 years, victim of abuse).

*“Violence is always from someone stronger over someone weaker, and often, the more vulnerable people do not react because they know they are dependent.”* (Male, 60–64 years, victim of abuse).

*“Because they (older people) are weak, they cannot do anything, and many of them are even subservient to their children or people who take care of them, just because they have a little bit of money and then they help, if they do not have money they beat them, it’s a betrayal of trust, that’s all there is to it.”* (Male, 60–64 years, victim of abuse).

### How respondents feel about elder abuse: revolt, anger and ingratitude

The phenomenon of elder abuse provokes feelings of revolt and powerlessness in those interviewed. In their view, it is also an expression of ingratitude on the part of the perpetrators towards those who have taken care of them throughout their lives.

*“I feel disgusted and I think about things I should not think about. I think old people should have a defence. They do not.”* (Male, 75–84 years, non-victim).

*“I feel the same revolt when I know that parents are abusing their children and when we see the terrible news that there is more domestic violence.”* (Male, 60–64 years, victim of abuse).

*“I am depressed! My husband says: ‘You’re sick, do not watch the news (about elder abuse).”* (Female, 65–74 years, victim of abuse).

*“I am very sensitive! I rebel because, deep down, an older adult is like a child; he’s helpless. People are vulnerable and I feel a particular revolt when family members or other outsiders do that, that is, use violence against older people.”* (Female, 65–74 years, victim of abuse).

*“I do not need to be attacked physically when I’m attacked. But ingratitude hurts me the most when I’m attacked verbally or gesturally by someone for whom I’ve given everything and more. What hurts me most is ingratitude. Is ingratitude violence? Undoubtedly there is more ingratitude today than in the past because in the past, even in the cities, people lived, were born and died at home. Today, people are born outside and die outside; if they fail at home, they are immediately thrown out.”* (Male, 75–84 years, non-victim).

Finally, while recognising that abuse of older people is timeless, respondents felt that older people were more protected and respected in their parents’ generation, unlike in today’s societies.

*“In my parents’ time, I do not remember any violence against the old people. On the contrary, there was a lot of protection for old people.”* (Male, 81, non-victim).

*“Society was more interventionist. The old society did not tolerate what is happening today.”* (Male, 65–74 years, victim of abuse).

## Discussion

The findings reported in this article focus on respondents’ perceptions of the status of older people in contemporary society, their perceptions of what constitutes elder abuse, what the main forms of violence against older people are, why they are particularly vulnerable to abuse, and how they feel about the phenomenon. Thus, we found optimistic discourses on ageing among the interviewees, influenced by the concept of active and healthy ageing. They see longevity as an opportunity to socialise with the family and engage in leisure and other activities they did not have the chance to do when they were younger (Martinson and Berridge, 2015).

The discourse of active and healthy ageing is strongly promoted by international institutions (e.g., WHO). However, in a review of the literature on this concept, Martinson and Berridge (2015) show that it focuses only on individual success, i.e., it puts the onus on individuals to manage risk at the level of preventing disease and declining with age, minimising the structural factors that decisively influence ageing in society. They also point out that the concept of active and healthy ageing fails to take into account the diversity of social and economic contexts in which ageing takes place and that the binary of successful and unsuccessful ageing (with illness and functional and psychological disability) ultimately leads to the development of new age discrimination or polarised age discrimination (Martinson and Berridge, 2015). Indeed, we also find a negative discourse among the interviewees that associates old age with illness, dependence and discrimination, and stereotypical notions of ageing that translate into negative and hostile attitudes towards older people. Those interviewed internalise these ageist notions of ageing and use them in their discourses to justify the multiple discrimination and violence to which they are subjected (Liang and Luo, 2012; Dias et al., 2022).

This finding has also been illustrated in other studies that relate the abuse of older people to the representations of age that exist in our societies. Calasanti (2005) and Nelson (2005) suggest that older people are stigmatised and socially excluded based on their age; Harbison (1999) links elder abuse to the reverence for youth and devaluation of older adults that exists in our time; Nahmiash (2002) suggests that elder abuse is linked to disdain for ageing, social rejection of older people and the entrenched belief that older adults are a burden. Crichton et al. (1999) report that age prejudice makes older adults vulnerable to abuse, and Podnieks (2006) cites ageism as a factor in the underreporting of elder abuse. Ward (2000) suggests that age bias is ingrained in our social institutions. This may explain why the legal system in North America does not protect older people from fraud and other abuse. Ageing can also be cited as a reason why health services and social programmes for older adults are under-resourced compared to those for children and adolescents (Podnieks, 2006) and women victims of domestic violence.

Like our study, other studies have found results that reinforce the link between abuse and lack of family and economic support, dependency and disability, and the asymmetry of power between older people and perpetrators. In the same vein, Walsh et al. (2010), in a study they conducted in two Canadian cities, showed that older people’s vulnerability to abuse is very much related to experiences of oppression stemming from ageism, sexism, disability, racism, heterosexism, in short, the intersection of different types of oppression. According to the Walsh et al. (2010), the accumulation of oppressive factors creates a power imbalance that puts older people at risk of being assaulted and abused. It also leads to them being seen by society as weaker, dependent and different.

Respondents identified mental health conditions such as addiction and dementia as factors which make older people vulnerable to abuse. This finding is supported by several studies which show that older people are particularly at risk of elder abuse due to physical and mental disabilities (Brozowski and Hall, 2004) and a reduced ability to carry out activities of daily living (ADL) (Fulmer et al., 2005). Dementia has also been identified as a risk factor for elder abuse (Dyer et al., 2000; Fulmer et al., 2005). This risk increases with age. Depression has also been identified as a risk factor for elder abuse (Weerd and Paveza, 2005).

Research has shown that elder abuse occurs at micro, meso and macro levels (Nahmiash, 2002), with power and control playing a critical explanatory role (Montminy, 2005; Spangler and Brandl, 2007). Often mentioned by respondents and present in their definition of abuse is the power imbalance between older people and their perpetrators, the lack of family and community support, feelings of powerlessness and lack of control due to dependency. In addition, respondents view abuse as a crime and a violation of human rights (Sturdy and Heath, 2007). They are also of the opinion that there are different forms of abuse, with psychological abuse being one of the most serious forms. Therefore, in line with research in this area, the respondents’ perceptions and experiences of abuse are consistent. More specifically, they refer to elder abuse as any voluntary act that harms or is likely to harm an older adult or any omission that deprives older adults of the care they need for their well-being and the violation of their rights. Such acts occur in an interpersonal relationship where one expects to trust, care for, secure (live together) or depend on. The perpetrator may be a member of the family, a member of staff of an institution, a contracted care worker, a neighbour or a friend (Pritchard, 2007; Castle et al., 2015).

However, the respondents also consider elder abuse to be assault, robbery and theft in public spaces, forcing us to reflect on the possibility of broadening the concept of abuse itself. It also forces us to consider the varying norms, cultures and traditions of the social contexts in which the victims and perpetrators are situated (Castle et al., 2015).

It is also important to note that the experience of victimisation can influence respondents’ perceptions and how they define abuse, its different types and the purpose for which violence is used. For example, respondents who had been victims of abuse described the different types of abuse in more detail. They gave numerous examples of violent practices. They also linked the different types of violence, showing, as in the case of violence against women and children, that one form of violence rarely occurs in isolation. At the same time, perceptions of the cumulative occurrence of violence also have an impact on perceptions of the function or purpose of the use of a particular type of violence. For example, respondents’ perception that physical abuse is severe is linked to the perception that perpetrators often use violence for intimidation and financial exploitation of older people. This perception of physical violence as a means of coercing and intimidating victims for financial exploitation is not as common in, for example, domestic violence against younger victims as it is with older people.

In the case of sexual abuse, participants, especially those who have been abused, say that this type of violence includes rape and forced sex or inappropriate touching by strangers and their husbands. In fact, some of the women interviewed, having been socialised in a generation that was more repressive and controlled in terms of sexual practices, do not accept having active sexual relations with their husbands. Nor do they accept being touched in certain ways. This has an impact on their perception of sexual abuse in marital relationships in old age, as well as on the perception that this type of violence is one of the most serious forms of violence against older people, along with physical violence.

This finding on the perception of the severity of sexual abuse in old age also highlights the importance of considering gender and sexism as risk factors, as is the case with younger women who are victims of this type of violence (Mears, 2003; Hightower et al., 2013; Storey, 2020).

Walsh et al. (2010), for example, show in their study that female gender continues to accumulate oppressive factors in old age, putting more senior women at risk of being victims of various types of abuse, whether in the marital relationship or institutional settings, often with injuries as severe as those inflicted on younger women—black eyes, broken bones, burns, sexual assault, verbal and psychological aggression, etc. Although older men are also abuse victims, they report that sexist ideology puts women at greater risk of abuse than older men. At the same time, older men are physically better able to defend themselves in some situations, unlike older women (Mears, 2003; Hightower et al., 2013; Sandberg, 2013).

However, according to respondents, some types of abuse are typical of old age. These include financial exploitation by family and institutional settings, abandonment, depersonalisation, infantilisation, contempt, neglect and lack of attention and affection. Once again, respondents’ perceptions that they have no value in contemporary societies are closely linked to these representations of elder abuse. Negative images of being old, useless and worthless influence how they view abuse and how they feel about this social problem (Mysyuk et al., 2015, 2016). This discourse creates favourable conditions for the reproduction of abuse and its tolerance by victims and society. It is therefore important to recognise elder abuse as a specific form of violence that requires campaigns to combat it and policies that are adapted to the diversity of victim profiles, perpetrators and the social and institutional contexts in which it may occur (Jackson, 2016; Mysyuk et al., 2016).

The notion of ageing in terms of decline, dependency and deficit standardises the existential condition and the meaning of what it means to be old in our societies. Simultaneously, this view obscures the variables (such as social class, ethnicity, gender, education, occupation) that produce diverse and multiple identities among older people rather than single, stable ones. There is no universal ageing, so it is important to take into account the diversity of experiences at this stage of life, including experiences of abuse. Paying attention to the diversity of profiles and experiences of older people will allow professionals working in this field to discuss risk factors for abuse more broadly and increase the effectiveness of intervention campaigns that better target the needs of victims (Mysyuk et al., 2016; Storey, 2020).

This discussion has also taken place in the context of investigations into the abuse of children. In the case of elder abuse, however, we are dealing with adults who have a different social status and a long experience of life. Elder protection policies are aimed at adults who are potentially aware of their rights, which makes it more difficult for professionals to intervene. Unlike children, most older people in our societies are independent and can make decisions about the support and care they wish to receive. Interventions must be specific and recognise that they are aimed at adult citizens with their own needs (Brogden and Nijhar, 2013).

Finally, when faced with the phenomenon of elder abuse, respondents report feelings of anger, revolt and psychological stress. However, they also believe it is a matter of ingratitude on the part of family members and society, which despises their role (Comijs et al., 1998; Dong et al., 2013).

## Limitations and future research directions

For the HARMED study, only cognitively able older adults were recruited (Folstein et al., 1975). Therefore, the results of this study cannot be extrapolated to other populations, such as people living in nursing homes. This is because the sample consists only of older participants who are integrated into the community.

However, the main aim of this article has been to provide, through the perceptions and discourses of the participants, some dimensions of analysis that allow an in-depth understanding of abuse and its tolerance by older people and society through the ideology of ageing. In-depth interviews have allowed older victims or non-victims of abuse to express themselves freely. Through this methodology, older people are listened to and can identify their perspectives and needs for preventing and combating the violence they are victims of. In terms of future research, we also believe in the benefits of methodological triangulation, for example, using focus groups. This allows for sharing experiences of victimisation and the construction of collective visions of elder abuse. We also believe it is essential to compare older people’s perceptions with those of younger people about what constitutes abuse and its different forms. This approach will make it possible to work on perceptions, understand perspectives on the severity of each type of violence, and, in this way, provide clues for designing a more targeted social intervention to prevent every kind of violence and target each social category.

Analysing the violence experienced by older people in public spaces, which they also identify as a form of elder abuse, would be another direction for future research.

## Conclusion and implications

The results of this study underline the need for research into older people’s perceptions of abuse and what they perceive to be the most common and serious types of abuse they have been subjected to. A key finding of this article is that respondents’ negative perceptions of old age and ageing in our societies impact their perceptions of violence against older people and the reasons why it is perpetrated. Thus, not only do the interviewees’ discourses reproduce negative social images of older adults as worthless, useless, dependent, sick and with physical and cognitive disabilities, but they also show their belief that the abuses are somehow allowed by the norms of contemporary society, which encourages the growth of ageist ideology instead of combating it. These negative images of what it means to be old in today’s societies cannot be countered by the social discourse on active and healthy ageing. On the contrary, respondents believe that current culture and norms are complicit in the increasing violence against older people. They feel helpless and have no control over these situations. They feel a kind of learned helplessness (Maier and Seligman, 1976), which means that no one is able to help them. This is exacerbated by their perception that there is currently less support from family and community.

The verbalisation of this perception by some participants reveals the internalisation of ageist representations of old age as deficit and dependency, i.e., as a group of disempowered people, which creates a breeding ground for abuse. The deconstruction of these representations and the promotion of the ‘affirmative old age’ concept is therefore important (Sandberg, 2013). According to Sandberg (2013), affirmative old age takes into account the diversity of material and subjective experiences of ageing, as well as its symbolic and cultural dimensions. It also allows us to move beyond the binary concepts of dependent old age vs. active and healthy old age. Most importantly, it sees older people as agents of their own existence, capable of surviving and taking action, including against abuse (Rozanova, 2010; Chesney-Lind and Morash, 2013).

In the interviewees’ discourse, there is a strong perception that psychological, physical and financial abuse and neglect are the most common forms of violence against older people, especially in families and home care settings. However, in the interviewees’ discourse, we also identified their insecurity in public spaces, leading them to identify violent acts such as physical violence, assault and theft in public places and by strangers as elder abuse. It is, therefore, time to explore why older people consider this type of violence, perpetrated in public, to be a prevalent form of violence against them, similar to that in family relationships or nursing homes.

In conclusion, this study shows that elder abuse is a serious problem in Portugal but is less known and studied than other forms of violence, such as child abuse and violence against women. On the other hand, the studies that have been carried out have tended to favour quantitative and large-scale approaches (Fraga et al., 2014; Gil et al., 2015). Thus, one of the implications of the results presented in this article is the need to develop more qualitative studies to listen to older people, with or without having experienced victimisation, to understand their feelings and perceptions about the variety of acts that they classify as abuse. This is useful because it makes it possible to develop further and extend the analytical and classificatory capacity of the concept of elder abuse, and thus to direct better the attention of intervention strategies towards specific acts of abuse that are not considered as such by health and criminal justice authorities. It also allows medical and social services to consider the diversity of perpetrators and the different social contexts in which elder abuse occurs.

The data also show that abuse rarely occurs alone, like other forms of domestic violence. The difference is that there is a perception among our respondents that some forms of violence are used as an ally to other forms of violence. For example, to make it easier for perpetrators to control the finances and property of older people, physical violence is used as an intimidation tool. Awareness of these perceptions by health, social and justice professionals is essential to prevent physical abuse and financial exploitation by both familiar and unfamiliar people.

Finally, this article highlights the need for the perspectives and needs of older people to be taken into account in the design of social policies to prevent violence. This will improve the targeting of policies and their effectiveness in preventing and controlling this public health problem. The article also highlights the need to strengthen social support for older people to improve violence monitoring strategies and the resilience of the ageing population.

## Data availability statement

The raw data supporting the conclusions of this article will be made available by the authors, without undue reservation.

## Ethics statement

The studies involving humans were approved by the joint ethics committee of the S. João Hospital and the Medical School of the University of Porto approved this study protocol (CES-320/2016). The studies were conducted in accordance with the local legislation and institutional requirements. The participants provided their written informed consent to participate in this study.

## Author contributions

ID: Conceptualization, Data curation, Formal analysis, Funding acquisition, Investigation, Methodology, Writing – original draft. SF: Conceptualization, Funding acquisition, Investigation, Writing – review & editing.

## Footnotes

^1^Although this article does not address the main debates on the figure of the victim, our analyses include the influence of feminist victimology, which challenges individualistic and behavioural explanations of crime and addresses the structural inequalities that favour victimisation. It also advocates a critical victimology that looks at victimisation as a reflexive field of study, seeking to understand why certain actions are defined as criminal rather than others, and how intersecting vulnerability factors can facilitate abuse in old age (Valcore et al., 2021). Given these theoretical influences, and the fact that the victim can also be an agent and not always a passive victim (Chesney-Lind and Morash, 2013), this article sought to understand how experiences of victimisation undermine participants’ sense of justice and perceptions of the main forms of abuse experienced by older people in our society.
